# Intraorbital foreign body projectile as a consideration for unilateral pupillary defect

**DOI:** 10.1186/1865-1380-5-14

**Published:** 2012-03-05

**Authors:** Craig N Czyz, Thomas P Petrie, Jonathan D Harder, Kenneth V Cahill, Jill A Foster

**Affiliations:** 1 Division of Ophthalmology, Section Oculofacial Plastic and Reconstructive Surgery, Ohio University/OhioHealth Doctor's Hospital, 5100 W. Broad St., Columbus, OH 43228; 2 Department of Ophthalmology, Oral and Maxillofacial Surgery, Grant Medical Center, 111 South Grant Ave., Columbus, OH 43215; 3 Department of Ophthalmology, The Ohio State University, 410 W. 10th Ave., Columbus, OH 43210

## Abstract

Intraorbital foreign bodies are frequently the result of high-velocity injuries with varying clinical presentations. The resultant diagnosis, management, and outcome depend on the type of foreign body present, anatomical location, tissue disruption, and symptomatology. A patient who presented to the Emergency Department with a large intraorbital foreign body projectile that was not evident clinically, but found incidentally on computed tomography and subsequent plain films is reported. The emergency room physician needs to be aware of the differential diagnosis of a unilateral irregular pupil with or without visual acuity changes. The differential diagnosis for any trauma patient with an irregular pupil with significant visual loss must include intraorbital foreign body and associated injury to the optic nerve directly or via orbital compartment syndrome secondary to hemorrhage and/or edema. Patients with significantly decreased visual acuity may benefit from emergent surgical intervention. In patients with intact visual acuity, the patient must be monitored closely for any visual changes as this may require emergent surgical intervention.

## Background

Research has shown that 3% of all visits to the Emergency Department (ED) in the US are related to ocular trauma [1]. Rapid assessment and evaluation are imperative to preserve maximal visual function. However, close attention must also be paid to obtaining a detailed history from the patient, family member, or witness to the precipitating event. Intraorbital foreign body (IFB) is a term that refers to any foreign material within the bony orbit but outside of the globe [2]. Injuries involving IFBs are associated with high levels of ocular and orbital morbidity [3]. The majority of injuries involve young males, tool-related mechanisms, and metallic, non-organic materials [3,4]. Computed tomography (CT) is the best option for initial evaluation of an IFB; however, if metallic material is suspected a plane film may be of value [2,5,6].

Pupillary irregularity resulting from orbital trauma can be ocular or orbital in nature. Blunt or penetrating trauma to the globe can result in iris sphincter muscle disruption or disintertion of the iris from its origin [7]. Partial or complete disruption of cranial nerve III can also occur. The intraorbital anatomy of cranial nerve III contains a superior and inferior division. The superior division is responsible for innervation of the levator and superior rectus muscles. The inferior division supplies the medial and inferior rectus and inferior oblique muscles, as well as the parasympathetic fibers to the sphincter pupillae and ciliary muscles. Disruption of the inferior division can lead to a dilated pupil and/or decreased adduction and globe depression. Interruption of both divisions can lead to ptosis, mydriasis, and ophthalmoplegia. Slight compression of the pial blood vessels and the superficially located pupillary fibers could result in dilation of the pupil [8]. In a non-traumatic setting approximately 25% of cases of a unilateral dilated pupil are idiopathic [9].

## Case report

A 39-year-old Caucasian male lumber mill saw operator presented to the ED with the chief complaint of "I got hit in the face with a piece of wood." The initial triage report stated that there was no "significant" visual loss, left periorbital edema, and no other injuries. On initial evaluation the patient was noted to be alert and oriented, and to have "an isolated injury to the left eye and orbit area with normal neurologic exam." There was a "left fixed and dilated pupil with minimal extraocular motion," a "small amount of red blood in the anterior chamber," and "no light sensation whatsoever." An emergent ophthalmology consultation was placed, and a CT of the head and cervical spine was ordered. The CT of the head revealed artifact from a large metallic IFB. Emergent oculoplastics and neurosurgery consults were then ordered.

The patient expanded on the history, adding that he had been using a saw consisting of a large metal disc with smaller C-shaped blades bolted onto it when something flew off and hit him on the left side of the face. He denied loss of consciousness but did describe pain in the left orbital area. He again reiterated that initially he was able to see out of the affected eye, but his vision went away quickly until he was unable to see anything. The patient denied having any significant previous ocular history.

Upon ophthalmologic examination, the patient had an uncorrected Rosenbaum near card visual acuity of J1+ (20/20) in the right eye and no light perception in the left eye. Intraocular pressures were 15 mmHg in the right eye and 17 mmHg in the left by Perkins tonometer. Extraocular motility was full in the right eye and severely reduced in all directions of gaze in the left eye. The right pupil was 3 mm and constricted to 2 mm with direct light. The left eye had a 7-mm pupil that did not react to direct or consensual light. A "swinging-flashlight test" confirmed a reverse afferent pupillary defect in the left eye. Confrontation visual fields were full in the right eye and not present in the left eye. External, slit lamp, and fundoscopic examination of the right eye was unremarkable.

**Figure 1:**
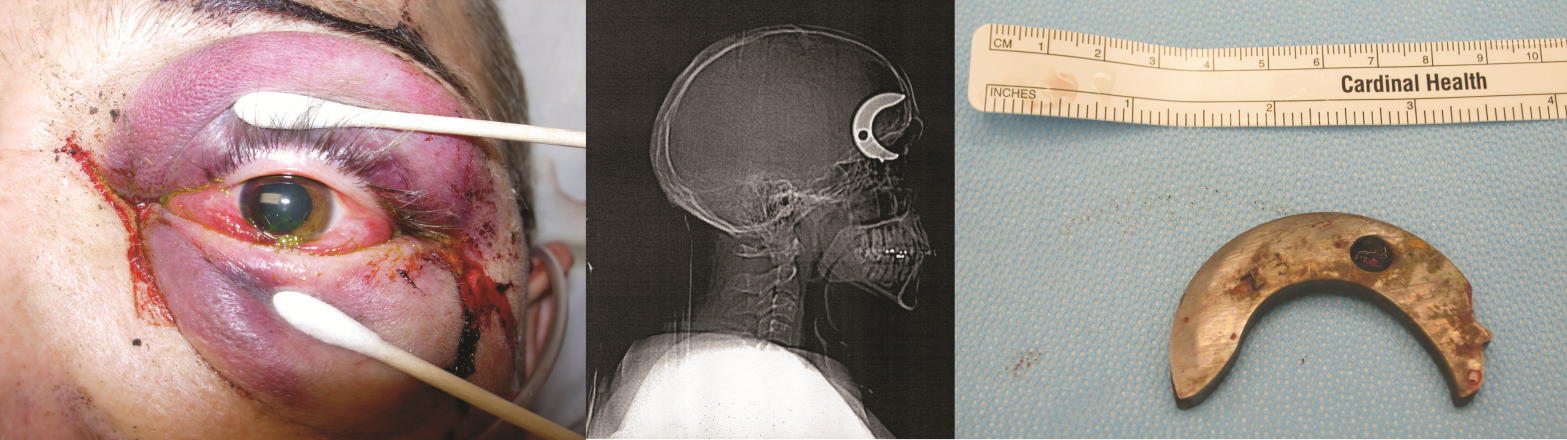
**a *Left*: External photograph of left eye and periorbital area**. **Note the laceration lateral and inferior to the lateral canthus. **b ***Center*: Sagittal plain film radiograph of the head and cervical spine shows a large sickle-shaped metallic density extending through the left orbital roof into the left frontal lobe. **c**: *Right*: Metallic object shown in **b **immediately following surgical removal.**

External exam of the left eye revealed significant edema and ecchymosis of the upper and lower eyelids. There was a 1-cm laceration lateral and inferior to the lateral canthus (Figure 1a). Slit lamp examination of the left eye showed 360 degrees of conjunctival chemosis and hemorrhage. The cornea was clear without fluorescein uptake or Seidel sign. The anterior chamber was deep with 2+ cells and a 5% hyphema. The iris was round and without transillumination defect. The lens was well centered and clear. Direct and indirect fundoscopic examination of the left eye revealed a diffuse inferior vitreous hemorrhage. The optic nerve appeared pink and flat with a cup-to-disc ratio of 0.2. There were four optic disk hemorrhages present. The entire fundus appeared pale with diffuse intraretinal hemorrhages and a "cherry red" spot of the fovea. The vessels appeared normal throughout the fundus.

Plain film radiographs and CT of the head revealed a large sickle-shaped metallic density extending through the left orbital roof superiorly into the left frontal lobe (Figure 1b). Fragments of the orbital roof were displaced superiorly into the left frontal lobe. The metallic density was in close proximity to the left optic nerve without evidence of transection. Retrobulbar hemorrhage was noted with mild (2-3 mm) proptosis of the left globe. Additionally, there was a 10-mm left subdural hemorrhage on the left cerebral convexity.

The metallic IFB projectile was removed by neurosurgery and oculoplastics the next day. The item removed was a 3 × 0.25-inch solid steel, sickle-shaped saw component (Figure 1c). Postoperatively the patient continued to have no light perception vision in the left eye, and required further orbital surgery for worsening proptosis and ocular exposure with corneal decompensation. It was confirmed at the time of surgery that the entry point of the IFB was the 1-cm laceration inferior to the lateral canthus (Figure 1a).

## Conclusions

This case presents numerous teaching points of properly assessing periorbital/orbital injuries as well as an often overlooked differential diagnosis for unilateral pupillary defect. The clinical course of the patient underscores the importance of a detailed history and thorough examination. There are multiple "clues" within the history and presentation that point to an increased likelihood of an IFB. The patient's employment and mode of injury should raise suspicion for a potential IFB projectile. The small laceration adjacent to the lateral canthus was not documented during the ED evaluation, suggesting a penetrating injury was absent from the differential diagnosis.

The patient reported having acuity immediately following the injury with subsequent loss. This is suggestive for the onset of orbital compartment syndrome causing traumatic optic neuropathy, not transection of the optic nerve. Anatomically, posterior to the globe the extraocular muscles and their surrounding fascia form a conical structure encasing vital vessels, lymphatics, and the optic nerve sheath, which contains the ophthalmic artery and optic nerve [5.8]. Direct trauma or secondary compression to these structures may cause a dilated and fixed pupil. Patients can also sustain decreased acuity following the initial injury from a worsening hypehma or vitreous hemorrhage. While these were noted in this patient, their extent was not consistent with his level of acuity. It is likely the patient had orbital compartment syndrome secondary to retrobulbar hemorrhage and generalized tissue edema. In cases such as this, an emergent canthotomy/canthal cutdown may preserve visual function; however, time is imperative as permanent optic nerve injury can occur within minutes [10].

Patients who have sustained high-velocity periorbital or orbital injuries should be suspected of having an IFB. Their presentation ranges from being asymptomatic to varying degrees of visual disturbance and/or periorbital pain, edema, and ecchymosis. A detailed history and clinical examination are imperative as the severity of a penetrating orbital injury is often underestimated on physical exam [2]. Radiological studies are usually necessary to accurately assess the patient. Ultrasound can be useful but is contraindicated if there is the possibility of an open globe. Magnetic resonance imaging (MRI) can result in blindness if performed prior to ruling out the presence of a metallic foreign body. The best initial study to order is thin-section axial CT scans of the orbits with multiplanar reformation. MRI does have utility in detecting non-metallic organic foreign bodies that are often missed on CT or in the evaluation of optic nerve injuries, but it is not recommended as part of the initial evaluation [5,6]. Plain film radiographs do have use in grossly localizing an IFB that may be obscured by artifact on CT.

Surgical intervention in the patient with intraorbital foreign body is indicated in the presence of a sharp foreign body, signs of infection, proptosis, restricted motility, chemosis, palpable orbital mass, optic nerve compression, abscess, suspicion of organic material (wood, vegetable matter), fistula formation, presence of copper, or when adjacent structures are compromised. Inorganic foreign bodies that are asymptomatic and not easily accessible can sometimes be safely left in place [2,3,6].

## Abbreviations

IFB: intraorbital foreign body; ED: emergency department; CT: computed tomography; MRI: magnetic resonance imaging

## Consent

Written informed consent was obtained from the patient for publication and/or presentation of his medical data, including this case report and any accompanying images. A copy of the written consent is available for review by the Editor-in-Chief of this journal.

## Authors' information

JF is the president elect of the American Society of Ophthalmic Plastic and Reconstructive Surgery. JF and KC serve as section editors for the American Academy of Ophthalmology board review book series. JF is an oral examiner for the American Academy of Ophthalmology. KC is an oral examiner for the American Society of Ophthalmic Plastic and Reconstructive Surgery. CC is an assistant professor and Department Chair of Ophthalmology and Oculofacial Plastic and Reconstructive Surgery Section Head at Ohio University/Doctors Hospital. JF and KC are assistant professors of Ophthalmology at Ohio State University. CC, JF, and KC are all fellows of the American Academy of Ophthalmology and American Society of Ophthalmic Plastic and Reconstructive Surgeons. JF and KC are fellows of the American College of Surgeons, and CC is a fellow of the American College of Osteopathic Surgeons.

## Competing interests

The authors declare that they have no competing interests.

## Authors' contributions

CC was involved in direct patient care, concept, drafting, and critically revising the manuscript. TP was involved in drafting the manuscript and reviewing the pertinent literature. JH was involved in direct clinical care of the patient and aiding in drafting the manuscript. KC was involved in critically revising the manuscript for important intellectual content. JF was involved in critically revising the manuscript for important intellectual content. All authors read and approved the final manuscript.
